# A Chromosome-Level Reference Genome for the Black-Legged Kittiwake (*Rissa tridactyla*), a Declining Circumpolar Seabird

**DOI:** 10.1093/gbe/evad153

**Published:** 2023-08-17

**Authors:** Marcella Sozzoni, Joan Ferrer Obiol, Giulio Formenti, Anna Tigano, Josephine R Paris, Jennifer R Balacco, Nivesh Jain, Tatiana Tilley, Joanna Collins, Ying Sims, Jonathan Wood, Z Morgan Benowitz-Fredericks, Kenneth A Field, Eyuel Seyoum, Marie Claire Gatt, Don-Jean Léandri-Breton, Chinatsu Nakajima, Shannon Whelan, Luca Gianfranceschi, Scott A Hatch, Kyle H Elliott, Akiko Shoji, Jacopo G Cecere, Erich D Jarvis, Andrea Pilastro, Diego Rubolini

**Affiliations:** Department of Biology, University of Florence, Sesto Fiorentino, Florence, Italy; Department of Environmental Science and Policy, University of Milan, Milan, Italy; Vertebrate Genome Laboratory, The Rockefeller University, New York, New York, USA; Department of Biology, Queen’s University, Kingston, Ontario, Canada; Department of Biology, The University of British Columbia, Kelowna, British Columbia, Canada; Department of Life and Environmental Sciences, Marche Polytechnic University, Ancona, Italy; Vertebrate Genome Laboratory, The Rockefeller University, New York, New York, USA; Vertebrate Genome Laboratory, The Rockefeller University, New York, New York, USA; Vertebrate Genome Laboratory, The Rockefeller University, New York, New York, USA; Tree of Life, Wellcome Sanger Institute, Cambridge, United Kingdom; Tree of Life, Wellcome Sanger Institute, Cambridge, United Kingdom; Tree of Life, Wellcome Sanger Institute, Cambridge, United Kingdom; Department of Biology, Bucknell University, Lewisburg, Pennsylvania, USA; Department of Biology, Bucknell University, Lewisburg, Pennsylvania, USA; Department of Biology, Bucknell University, Lewisburg, Pennsylvania, USA; Department of Environmental Science and Policy, University of Milan, Milan, Italy; Department of Natural Resource Sciences, McGill University, Ste-Anne-de-Bellevue, Quebec, Canada; Centre d’Études Biologiques de Chizé (CEBC), UMR 7372 - CNRS & Université de La Rochelle, Villiers-en-Bois, France; Department of Life and Environmental Science, University of Tsukuba, Tsukuba, Japan; Department of Natural Resource Sciences, McGill University, Ste-Anne-de-Bellevue, Quebec, Canada; Department of Biosciences, University of Milano, Milan, Italy; Institute for Seabird Research and Conservation, Anchorage, Alaska, USA; Department of Natural Resource Sciences, McGill University, Ste-Anne-de-Bellevue, Quebec, Canada; Department of Life and Environmental Science, University of Tsukuba, Tsukuba, Japan; Area Avifauna Migratrice, ISPRA, Ozzano dell’Emilia, Italy; Vertebrate Genome Laboratory, The Rockefeller University, New York, New York, USA; Howard Hughes Medical Institute, Chevy Chase, Maryland, USA; Department of Biology, University of Padova, Padova, Italy; Department of Environmental Science and Policy, University of Milan, Milan, Italy; Water Research Institute, IRSA-CNR, Brugherio, Monza and Brianza, Italy

**Keywords:** Arctic, gull, population decline, chromosome-level genome assembly, Charadriiformes, chromosomal rearrangements

## Abstract

Amidst the current biodiversity crisis, the availability of genomic resources for declining species can provide important insights into the factors driving population decline. In the early 1990s, the black-legged kittiwake (*Rissa tridactyla*), a pelagic gull widely distributed across the arctic, subarctic, and temperate zones, suffered a steep population decline following an abrupt warming of sea surface temperature across its distribution range and is currently listed as Vulnerable by the International Union for the Conservation of Nature. Kittiwakes have long been the focus for field studies of physiology, ecology, and ecotoxicology and are primary indicators of fluctuating ecological conditions in arctic and subarctic marine ecosystems. We present a high-quality chromosome-level reference genome and annotation for the black-legged kittiwake using a combination of Pacific Biosciences HiFi sequencing, Bionano optical maps, Hi-C reads, and RNA-Seq data. The final assembly spans 1.35 Gb across 32 chromosomes, with a scaffold N50 of 88.21 Mb and a BUSCO completeness of 97.4%. This genome assembly substantially improves the quality of a previous draft genome, showing an approximately 5× increase in contiguity and a more complete annotation. Using this new chromosome-level reference genome and three more chromosome-level assemblies of Charadriiformes, we uncover several lineage-specific chromosome fusions and fissions, but find no shared rearrangements, suggesting that interchromosomal rearrangements have been commonplace throughout the diversification of Charadriiformes. This new high-quality genome assembly will enable population genomic, transcriptomic, and phenotype–genotype association studies in a widely studied sentinel species, which may provide important insights into the impacts of global change on marine systems.

**Significance**

The integration of genomic data with environmental and ecological data is shedding light on the effects and responses of animal populations to global changes, including climate change. Here, we present a chromosome-level reference genome for the black-legged kittiwake, a pelagic gull species with a circumpolar distribution in the northern hemisphere that has experienced population declines because of the abrupt warming of sea surface temperatures in recent decades. This chromosome-length genome assembly is an important resource for future genomic studies aimed at evaluating the impacts of climate change in a prime indicator species of fluctuating conditions in marine ecosystems.

## Introduction

Over the last few years, the generation of chromosome-level genome assemblies has steadily increased across the tree of life (Lewin et al. 2018; Rhie et al. 2021; Formenti, Theissinger, et al. 2022), allowing us to better characterize genome structure and function. Highly contiguous genomes, especially when used in combination with whole-genome resequencing data and high-quality annotations, enable an accurate characterization of large structural variants (Mérot et al. 2020; Shingate et al. 2020; Matschiner et al. 2022); the study of chromosome evolution (Huang et al. 2022); and accurate inferences of past demographic trends, levels of inbreeding, deleterious variants, and genomic erosion in populations of conservation concern (Dussex et al. 2021; Bertorelle et al. 2022; Kardos et al. 2023).

Gulls have been the subject of intensive research in behavior, physiology, ecotoxicology, and ecology (Tinbergen and Perdeck 1951; Oro et al. 1999; Kitaysky et al. 2001; Goutte et al. 2015). However, evolutionary genomics studies have been hampered by the scarcity of available genomic resources in this lineage. To date, only 4 of the 53 currently recognized gull species have reference genomes (Feng et al. 2020; Yang et al. 2021), with none of these consisting of high-quality chromosome-level genome assemblies.

The black-legged kittiwake (*Rissa tridactyla*; hereafter “kittiwake”) is a small pelagic gull that breeds in arctic and subarctic zones across the Northern Hemisphere (Coulson 2011). The two recognized subspecies, *R. t. tridactyla*, distributed across the Atlantic and Arctic basins and *R. t. pollicaris* occurring in the North Pacific, differ in terms of morphology and life-history traits (Hatch et al. 1993) and are genetically differentiated based on mitochondrial DNA analyses (Sauve et al. 2019). Kittiwakes have been the subject of several intensive long-term monitoring programs in the United Kingdom, France, the United States, and Norway (Aebischer and Coulson 1990; Hatch et al. 1993; Jacobsen et al. 1995; Danchin et al. 1998), where they are considered prime indicators of fluctuating conditions in marine ecosystems (Frederiksen et al. 2007; Wanless et al. 2007). For example, kittiwakes have experienced steep population declines coinciding with abrupt warming of sea surface temperatures in the 1990s (Descamps et al. 2017). Although it remains the most abundant gull species in the world, with an estimated global population size of 14,600,000–15,700,000 individuals (Wetlands International 2015), the kittiwake has been listed as vulnerable by the International Union for the Conservation of Nature Red List since 2017 (Birdlife International 2019) following steep population declines. Here, we present the first chromosome-level, annotated and haplotype-resolved reference genome for the kittiwake; we compare it with a previous assembly based on short reads (Feng et al. 2020) and perform pairwise whole-genome alignments across four chromosome-level Charadriiformes genomes and two avian outgroups to uncover chromosomal rearrangements.

## Results and Discussion

### Genome Sequence Statistics

**Fig. 1.**
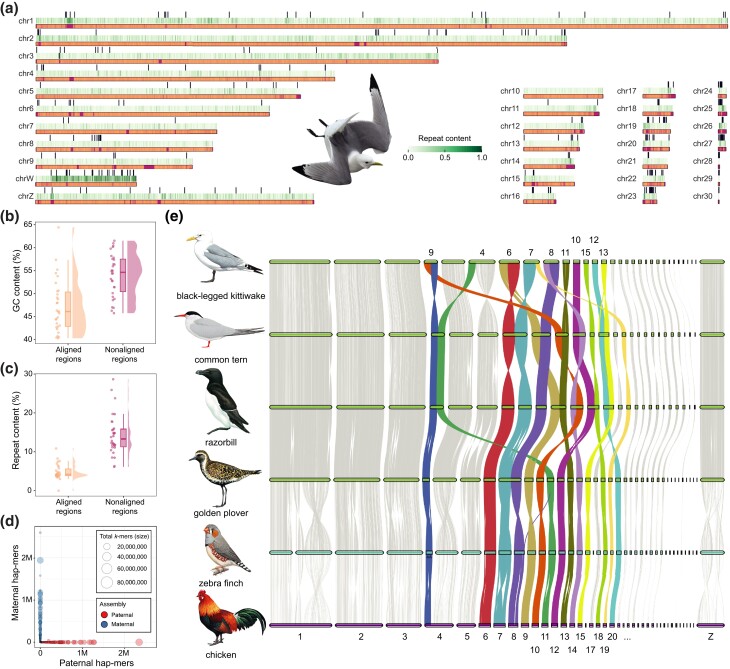
A chromosome-level genome assembly for the black-legged kittiwake (bRisTri1) and interchromosomal rearrangements across Charadriiformes. (*a*) Alignment of bRisTri1 and OUT-0021 genome assemblies. For each chromosome, the bottom track represents the regions of bRisTri1 chromosomes that aligned (peach) or did not align (violet) to a sequence of the OUT-0021 assembly. The middle track represents the repeat content in windows of 1 kbp, with darker green representing higher repeat content as shown in the legend. The top track shows the assembly gaps as black bars. (*b*) Raincloud plots showing the GC content in aligned and nonaligned regions between the two assemblies. (*c*) Repeat content in the same regions. (*d*) Hap-mer blob plot of the bRisTri assembly. Each blob represents a contig, and its size is proportional to contig size. Red blobs represent maternal haplotype contigs, whereas blue blobs represent paternal haplotype contigs. Each blob is plotted according to the number of paternal and maternal hap-mers that it contains. The fact that there are very few paternal-specific *k*-mers in the maternal assembly and vice versa suggests that each contig was successfully phased. (*e*) Pairwise whole-genome alignments across four chromosome-level Charadriiformes genomes (black-legged kittiwake, common tern, razorbill, and European golden plover) and two outgroups (zebra finch and chicken). Horizontal bars represent chromosomes and are colored in green for the Charadriiformes, turquoise for the zebra finch, and purple for the chicken. Chromosomes are ordered by the chicken chromosome IDs, which are labeled at the bottom of the figure. Black-legged kittiwake chromosome IDs for chromosomes that have experienced interchromosomal rearrangements in the Charadriiformes are labeled at the top, and these chromosomes are highlighted in colors. Bird illustrations reproduced with permission by Lynx Edicions.

We produced the first chromosome-level and fully phased reference genome (hereafter “bRisTri1”) for the kittiwake using the Trio Vertebrate Genomes Project (VGP) genome assembly pipeline 2.0. Phasing was aided by the use of parental sequence data, where we obtained two phased assemblies, one for each parental haplotype (fig. 1*d* and supplementary fig. S1, Supplementary Material online). After manual curation (supplementary fig. S2, Supplementary Material online), the paternal haplotype was selected as the reference genome because it was the most contiguous, and the maternal chromosome W was added to the paternal assembly for completeness. Manual curation increased the scaffold N50 by 16.99% (from 75.3 to 88.2 Mb) and reduced the scaffold count by 8.93% (from 784 to 714). The curated assembly has an assembly span of 1.35 Gb, which is slightly longer than the *k*-mer assessment (1.20 Gb; supplementary fig. S3, Supplementary Material online), a per base consensus quality value of 62.39 (0.58 base errors/Mb), and a BUSCO completeness score of 97.4% (table 1). We assigned 91.82% of the assembled sequence to 30 autosomes (named according to decreasing size) and to the Z and W sex chromosomes. We also obtained a complete and circularized mitochondrial genome (16,835 bp) using the maternal short-read data. The new mitogenome includes the whole control region, which was not included in the current NCBI reference sequence for the species (MN356420.1; supplementary fig. S4, Supplementary Material online).

**Table 1:** Comparison of Assembly Statistics, BUSCO Results, and Annotation Statistics between the bRisTri1 (This Assembly) and the OUT-0021 (Previous Assembly) Black-Legged Kittiwake Genome Assemblies

Assembly Statistics	bRisTri1 (VGP)	OUT-0021 (B10K)
Genome size (Gb)	1.35	1.19
Number of chromosomes	32	—
Number of scaffolds	716	53,283
N50 (Mb)	88.21	17.65
L50	5	20
BUSCO assembly (aves_odb10)	C: 97.4% (S: 96.7%, D: 0.7%), F: 0.5%, M: 2.1%	C: 97.2% (S: 96.9%, D: 0.3%), F: 0.6%, M: 2.2%
BUSCO annotation (aves_odb10)	C: 99.0% (S: 98.3%, D: 0.7%), F: 0.2%, M: 0.8%	C: 81.9% (S: 81.5%, D: 0.4%), F: 5.7%, M: 12.4%
Genes and pseudogenes	19,635	16,143
Protein-coding genes	16,534	14,776
Repeat content (%)	8.61	8.70

### Genome Annotation

The NCBI Eukaryotic Genome Annotation pipeline identified 19,635 genes and pseudogenes, including 16,534 protein-coding genes (table 1). A BUSCO analysis of the annotation resulted in 99.0% complete BUSCO genes (single copy: 98.3%, duplicated: 0.7%). BLASTP results against the UniProtKB/Swiss-Prot–curated proteins showed that 97.6% of genes had a protein with an alignment score covering 50% or more of the query and 68.5% had an alignment covering 95% or more of the query (see NCBI annotation report https://www.ncbi.nlm.nih.gov/genome/annotation_euk/Rissa_tridactyla/GCF_028500815.1-RS_2023_03/).

The de novo repeat annotation showed that 8.61% of the genome consists of repetitive regions (supplementary table S1, Supplementary Material online), which is within the range of previously sequenced bird genomes (Zhang et al. 2014; Feng et al. 2020).

### Comparison with Previous Genome Assembly

We compared the bRisTri1 assembly with a previous kittiwake draft genome assembly generated using short-read data (OUT-0021; Feng et al. 2020). The OUT-0021 assembly was produced using a short-insert size library from an individual from Prince Leopold Island (Nunavut, Canada; subspecies *tridactyla*) and three long-insert size libraries (used for scaffolding) from an individual from Middleton Island (AK, USA; subspecies *pollicaris*). Thus, the OUT-0021 assembly sequence is representative of the *tridactyla* subspecies but could present genome assembly incongruences because of potential genome structure divergence between the two subspecies. Conversely, the bRisTri1 assembly was produced using data from a single individual from Middleton Island and its parents and therefore represents both the genome sequence and the structure of the *pollicaris* subspecies.

Our reference genome has a longer assembly length than the previous assembly (bRisTri1 = 1.35 Gb vs. OUT-0021 = 1.19 Gbp) and a higher scaffold N50 (bRisTri1 = 88.21 Mb vs. OUT-0021 = 17.65 Mb) (table 1). Despite the higher contiguity of the bRisTri1 assembly, genome completeness based on BUSCO analysis was only marginally higher in bRisTri1 (97.4%) than in OUT-0021 (97.2%). However, when we assessed the presence of BUSCOs in the annotations for the two assemblies, BUSCO completeness was much higher for the bRisTri1 annotation (99% vs. 81% for OUT-0021). Indeed, the bRisTri1 annotation contained 1,758 more protein-coding genes and 3,492 more genes and pseudogenes than the OUT-0021 annotation.

To allow for an efficient comparison of the two genome assemblies, we performed a pairwise whole-genome alignment (fig. 1*a*). The regions in bRisTri1 that did not have an OUT-0021 alignment (purple sections in fig. 1*a*, total length = 311 Mb) showed a higher GC content (Mann–Whitney *U* test, *W* = 153, *P* = 4.1 × 10^−6^; fig. 1*b*) and a higher repeat content (Mann–Whitney *U* test, *W* = 17, *P* = 2.1 × 10^−14^; fig. 1*c*) than the aligned regions (peach sections in fig. 1*a*, total length = 1,042 Mb), consistent with the fact that GC-rich and repeat-rich regions are challenging to assemble using short-read data (Botero-Castro et al. 2017; Kim et al. 2022).

### Chromosomal Rearrangements in Charadriiformes

Cytogenetic studies have shown that Charadriiformes display a highly variable number of chromosome pairs (40–94; Degrandi et al. 2020). To explore the occurrence of chromosomal rearrangements in this lineage at a finer scale, we aligned the chromosome-level assemblies of four Charadriiformes species (black-legged kittiwake, common tern [*Sterna hirundo*], razorbill [*Alca torda*], and European golden-plover [*Pluvialis apricaria*]) and two outgroups (chicken [*Gallus gallus*] and zebra finch [*Taeniopygia guttata*]; fig. 1*e*).

We observed only two interchromosomal rearrangement events (both fissions) between the chicken and the zebra finch related to chicken chromosomes 1 and 4. In sharp contrast, multiple chromosomal fusions or fissions seem to have occurred in Charadriiformes (fig. 1*e*). We found that no fissions or fusions are shared among the Charadriiformes genomes we analyzed, except for the razorbill and the common tern sharing two fusion events (chicken Chr4 + Chr11 and Chr15 + Chr20). All remaining observed rearrangements are specific to the lineages leading to each sampled species, a pattern that was also recently evidenced in parrots (Huang et al. 2022).

The frequency of rearrangements varies among chromosomes, with some having undergone numerous and independent (mostly fusion) rearrangements. Ten chromosomes have undergone at least two chromosomal rearrangements in Charadriiformes (chicken Chr4, Chr6, Chr7, Chr8, Chr9, Chr10, Chr11, Chr12, Chr13, and Chr14). For example, Chr7 is fused with Chr8 in the golden plover, to Chr6 in the common tern, and to Chr19 in the kittiwake (fig. 1*e*). Similar to what has been shown in parrots, we found that intermediate-sized chromosomes (20–40 Mb) were more frequently rearranged, suggesting that these chromosomes may be more prone to rearrangements across birds. To confirm that the inferred chromosomal rearrangements were not due to misassemblies, we evaluated the kittiwake Hi-C contact maps, which confirmed every inferred chromosomal rearrangement (supplementary fig. S2, Supplementary Material online). Interestingly, chromosome fissions and fusions occur more frequently in groups with higher speciation rates (Leaché et al. 2016; de Vos et al. 2020), possibly because such rearrangements act as barriers to gene flow (Mackintosh et al. 2023). Our results suggest that chromosomal rearrangements might be important for speciation in the diverse- and species-rich Charadriiformes and are in contrast to the view that a low rate of chromosomal rearrangements in birds might be the reason for a slow accumulation of postzygotic incompatibilities in birds (Ellegren 2010).

## Conclusions

The new chromosome-level genome for the kittiwake is significantly less fragmented than the previous draft assembly and includes 160 Mb of extra sequence enriched in high GC content and high repeat content regions. By performing pairwise whole-genome alignments across four chromosome-level Charadriiformes genomes, we uncovered several lineage-specific chromosome fusions and fissions, suggesting that such events may be important for the evolution of this species-rich group. This genome assembly will enable the mapping of population genomic, transcriptomic, and phenotype–genotype association data, allowing a better understanding of the mechanisms underpinning the broad physiological and behavioral plasticity of the kittiwake. Moreover, such analyses will allow researchers to better explore the effects of past climatic oscillations on the demographic history of this indicator species of fluctuating conditions in marine ecosystems. These future analyses will provide important insights into the impacts of global change on animal communities inhabiting rapidly changing regions.

## Materials and Methods

### Sampling, DNA Extraction, and Sequence Data Generation

Blood samples (100 μl) were extracted from the brachial vein of three kittiwake individuals (subspecies *pollicaris*) and were stored at −20 °C. We sampled a female offspring (USGS band number: 79426718) and its parents (USGS band numbers: 79426635 and 79426718), to obtain both sex chromosomes. For RNA sequencing, we collected a pectoralis muscle biopsy from an additional male (USGS band number: 1064-04595), which was preserved in RNAlater 1:5 at −20 °C for 2 weeks and then at −80 °C until RNA extraction. All samples were collected in Middleton Island, Alaska (59.48 N, 146.38 W), between 2010 and 2016 under license from the US Fish and Wildlife Service.

Genomic data for assembly were generated using three different sequencing technologies: Pacific Biosciences (PacBio) HiFi long-read sequencing, Bionano optical maps with one restriction enzyme (Direct Label and Stain) labeling, and Hi-C contact maps from Arima Genomics v2 chemistry. DNA was extracted using Bionano Prep SP Frozen Human Blood DNA Isolation protocol v2 (modified for nucleated blood extractions). DNA was sheared on the Megaruptor 3 (Diagenode, Denville, NJ, USA) before PacBio library preparation. The HiFi SMRTbell® library was prepared with the SMRTbell Express Template Prep Kit 2.0 following the manufacturer's protocol (Pacific Biosciences, Menlo Park, CA, USA) and size selected thereafter on a Pippin HT (Sage Science, Beverly, MA, USA) with a 10 kb cutoff. The PacBio library was sequenced on a Sequel IIe (Pacific Biosciences, Menlo Park, CA, USA) with Sequencing Plate 2.0 and two 8 M SMRT cells. To generate Bionano optical maps, extracted DNA was labeled with DNA labeling enzyme (DLE-1), and its backbone was stained using the Bionano Prep Direct Label and Stain protocol before being loaded onto the Saphyr system. A blood aliquot was sent to the Arima Genomics sequencing facility to generate the Hi-C contact map data (Arima Genomics, Carlsbad, CA, USA).

For RNA sequencing, RNA was extracted from the muscle sample using Qiagen RNeasy Universal Plus Mini kit following the manufacturer's protocol (Qiagen, Hilden, Germany). RNA libraries were prepared using the NEBNext® Poly(A) mRNA Magnetic Isolation Module NEBNext® Ultra™ RNA Library Prep Kit for Illumina and sequenced on an Illumina NextSeq500 v2 platform using 150 bp PE sequencing at Admera Health (Plainfield, NJ, USA).

### Genome Size Estimation, Genome Assembly, and Assembly Evaluation

The genome was assembled using the VGP assembly pipeline (Rhie et al. 2021; Lariviere et al. 2023) on the online platform Galaxy Europe (Afgan et al. 2018). Prior to assembly, we generated a *k*-mer database from the HiFi reads using the software Meryl (1.3 + galaxy4; Rhie et al. 2020) with a *k*-mer size of 21. This database was then used to estimate genome size, abundance of repetitive elements, and heterozygosity rate using GenomeScope2 (2.0 + galaxy1; Vurture et al. 2017; Ranallo-Benavidez et al. 2020). The HiFi reads were then assembled using hifiasm (0.16.1 + galaxy2; Cheng et al. 2021) using the Trio mode, which employs parental reads to produce phased maternal and paternal assemblies. A first round of quality evaluation was performed using gfastas (1.2.0 + galaxy0; Formenti, Abueg, et al. 2022) and Merqury (1.3 + galaxy1; Rhie et al. 2020), and completeness was evaluated using BUSCO (5.2.2 + galaxy2; Simão et al. 2015). The assembly was first scaffolded with Bionano Optical maps (Bionano Hybrid Scaffold 3.7.0 + galaxy0; Shelton et al. 2015) and then with Hi-C reads using Salsa2 (2.3 + galaxy2; Ghurye et al. 2017, 2019). The complete assembly was then manually curated using gEVAL (Chow et al. 2016) and Hi-C maps (Howe et al. 2021). Contiguity and completeness were evaluated after every step of the genome assembly pipeline.

### Mitogenome Assembly and Annotation

Because of a depletion of mitochondrial long reads (see more details in supplementary text, Supplementary Material online), we assembled the maternal short reads using the de novo assembler Novoplasty 4.3.1 (Dierckxsens et al. 2017). The mitogenome was annotated using MITOS2 (Donath et al. 2019), and Proksee was used for visualization (Grant et al. 2023).

### Comparison with Previous Assembly

To compare the bRisTri1 assembly with the previously available assembly for the species (OUT-0021 B10K; Feng et al. 2020), we aligned the two genomes using nucmer from the MUMmer package (4.0.0cr1; Marçais et al. 2018). We extracted sequences longer than 1 kb (option -l 1000) present in both the reference (bRisTri1) and the query (OUT-0021 B10K) (option -mum). We used the tool show-coords from the MUMmer package to extract alignment coordinates for alignments with an identity of 98% or more (-I 98). Regions in the reference that had a query alignment and passed the identity filter were considered as aligned, and the rest of the regions was considered as unaligned. We then calculated the GC content and the repeat content in aligned and unaligned regions using bedtools nuc (v2.29.2, Quinlan and Hall 2010) in windows of 1 kbp. Alignments, proportion of repetitive sequence, and gaps were plotted using the package karyoploteR (Gel and Serra 2017).

### Genome Annotation and Repetitive Content

The bRisTri1 assembly was functionally annotated using the NCBI Eukaryotic genome annotation pipeline (Pruitt et al. 2014; Rhie et al. 2021). Both kittiwake assemblies were assessed for repetitive content using WindowMasker v.1.0.085 (Morgulis et al. 2006) and RepeatMasker v.4.1.086 (Smit et al. 2015) (http://www.repeatmasker.org). RepeatMasker was run with NCBI/RMBLAST 2.10.0+ with Dfam_3.1 (profile HMM library) and Repbase (Bao et al. 2015) version 20,170,127 as repeat databases using the “aves” repeat library.

### Pairwise Genome Alignments and Chromosomal Rearrangements

We used the MUMmer tool nucmer (4.0.0cr1, Marçais et al. 2018) with the option -b 400 to perform pairwise whole-genome alignments between the four species of Charadriiformes with chromosome-level genomes (black-legged kittiwake, common tern, razorbill, and golden plover; Rhie et al. 2021; Meyer et al. 2023) and the two outgroups (chicken and zebra finch; Rhie et al. 2021; Huang et al. 2023) and retained only the one-to-one alignments using delta-filter (option -1) from nucmer. Scaffolds not assigned to chromosomes were excluded from the analysis. Pairwise alignments were then formatted for input into the MCscan pipeline for synteny visualization (Tang et al. 2008).

## Supplementary Material

evad153_Supplementary_Data

## Data Availability

Paternal and maternal assemblies (bRisTri1) presented in this study are available on NCBI under accessions GCA_028501385.1 and GCA_028500815.1 along with NCBI *Rissa tridactyla* Annotation Release of the paternal haplotype GCF_028500815.1. The mitochondrial genome sequence is also available in NCBI under accession OR187459. All raw sequence data used to generate the genome assembly are archived to NCBI Sequence Read Archive under Bioproject PRJNA940128 and also on GenomeArk (https://genomeark.github.io/genomeark-all/Rissa_tridactyla.html).
