# Supplementary material for: A Chromosome-Level Reference Genome for the Black-Legged Kittiwake (Rissa tridactyla), a Declining Circumpolar Seabird
Source: Genome Biol Evol. 2023 Aug 17;15(8):evad153. doi: 10.1093/gbe/evad153 (PMC10457150; doi:10.1093/gbe/evad153)
Supplement: evad153_Supplementary_Data [file evad153_supplementary_data.pdf]

Supplementary Material for

A chromosome-level reference genome for the black-legged kittiwake (*Rissa tridactyla*), a marine top predator

Marcella Sozzoni, Joan Ferrer Obiol, Giulio Formenti, Anna Tigano, Josephine R. Paris, Jennifer R. Balacco, Nivesh Jain, Tatiana Tilley, Joanna Collins, Ying Sims, Jonathan Wood, Z. Morgan Benowitz-Fredericks, Kenneth A. Field, Eyuel Seyoum, Marie Claire Gatt, Don-Jean Léandri-Breton, Chinatsu Nakajima, Shannon Whelan, Luca Gianfranceschi, Scott A. Hatch, Kyle H. Elliott, Akiko Shoji, Jacopo G. Cecere, Erich D. Jarvis, Andrea Pilastro, Diego Rubolini

Corresponding author:
Joan Ferrer Obiol, Email: joan.ferrer.obiol@gmail.com

This pdf file includes:

Supplementary Text
Figures S1 to S4
Table S1

Supplementary Text

Mitogenome Assembly

To assemble the mitogenome, we first tested the long-read *de novo* assembler MitoHifi (Uliano-Silva et al. 2023) on the offspring PacBio data. However, the assembler failed to produce a mitochondrial sequence. Upon investigation, we identified that this was due to the depletion of mitochondrial long reads, as is frequently observed in such data sets (Formenti et al. 2021). We then tested the short-read *de novo* assembler Novoplasty 4.3.1. (Dierckxsens et al. 2017), using illumina data derived from both parents. This approach was also unsuccessful due to the presence of multiple putative NUMT copies covering ~70% of the mitochondrial sequence, which are likely to confound short-read based mitochondrial assembly. To confirm the presence of NUMTs, we mapped the short-read data to both the genome and the mitogenome concurrently. We inspected the location of reads that mapped to the mitogenome when only mapping to the mitogenome, but which mapped to the nuclear genome when mapping to both the genome and the mitogenome concurrently. This analysis revealed the presence of long NUMTs (~60% of the genome) in three different scaffolds (JAPUFG010000528.1, JAPUFG010000304.1 and JAPUFG010000101.1) and also smaller NUMTs in chromosome 1 and chromosome W. The NUMT reads were then filtered prior to another Novoplasty assembly, which recovered a circularised mitogenome. For quality control of the mitogenome assembly, we mapped the long reads to the mitogenome assembly, identifying two mapped reads spanning the whole mitogenome. The reads were merged using the EMBOSS merger tool (Rice et al. 2000) and the merged fragment was aligned to the mitogenome assembly. The two sequences were nearly identical, even in the control region which is absent in the NCBI mitochondrial reference sequence (MN356420.1) (Supplementary fig. S4). The only region where the two sequences significantly diverged was in a simple repeat region located between the control region and the tRNAPhe encoding gene. The simple repeat (CAA/CAAA) was 208 bp in length in the PacBio reads versus 128 bp in length in the assembly. This finding highlights the difficulty of accurately assembling low complexity regions with short-read data. Overall, our new mitogenome assembly for the kittiwake is more complete than the NCBI reference sequence (Supplementary fig. S4). This new assembly shows a 0.34% divergence from the current reference assembly, which belongs to subspecies *tridactyla*.

Literature Cited

Dierckxsens N, Mardulyn P, Smits G. 2017. NOVOPlasty: de novo assembly of organelle genomes from whole genome data. Nucleic Acids Res. 45:e18.

Formenti G et al. 2021. Complete vertebrate mitogenomes reveal widespread repeats and gene duplications. Genome Biol. 22:120.

Rice P, Longden I, Bleasby A. 2000. EMBOSS: the European Molecular Biology Open Software Suite. Trends Genet. 16:276–277.

Uliano-Silva M et al. 2023. MitoHiFi: a python pipeline for mitochondrial genome assembly from PacBio High Fidelity reads. bioRxiv. 2022.12.23.521667. doi: 10.1101/2022.12.23.521667.

Supplementary Figures

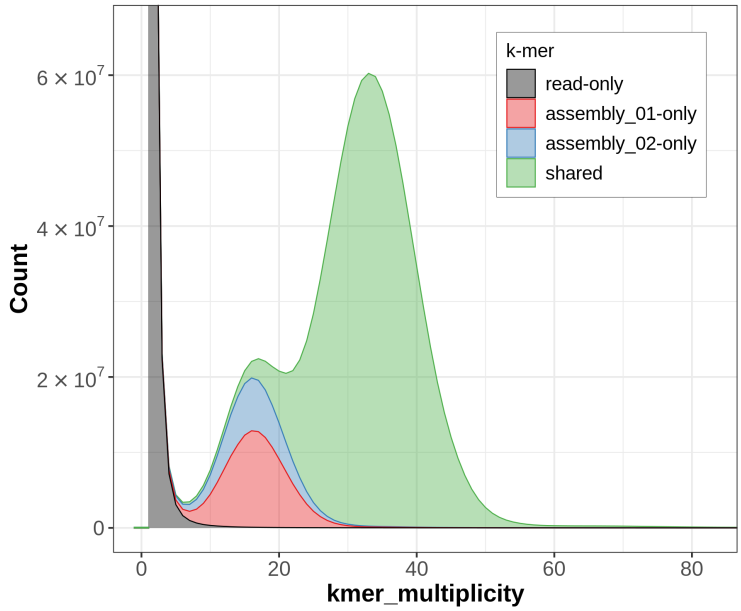

Figure S1. Merqury assembly spectra plot for evaluating *k*-mer completeness. *K*-mers are coloured by their presence: grey represents *k*-mers present only in the reads; red represents *k*-mers present, only in the maternal assembly; blue represents *k*-mers present only in the paternal assembly; and green represents *k*-mers present in both assemblies.

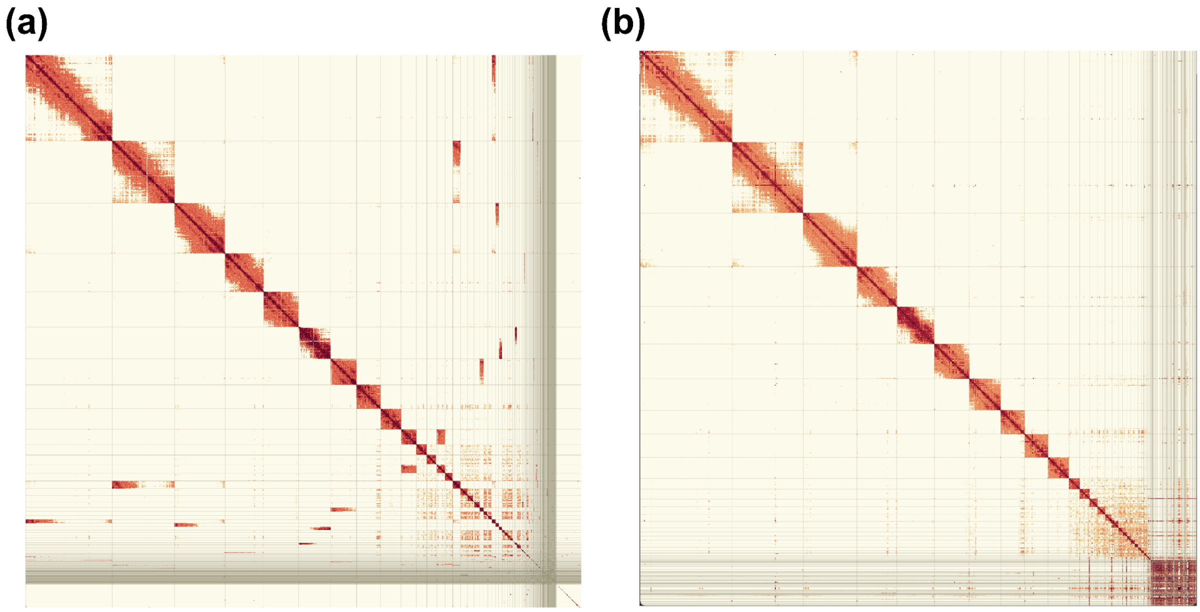

Figure S2. Hi-C interaction heatmaps for the bRisTri1 assembly before (a) and after manual curation (b). Both axes represent the linear sequence of the reference genome and the frequency at which two DNA fragments physically associate in 3D space is shown by the colour intensity, with more intense colours representing a higher physical association. A few off-diagonal interactions are visible before curation (a), which represent missing links between scaffolds of the same chromosome or misassemblies. These off-diagonal interactions are mostly removed in the curated assembly (b).

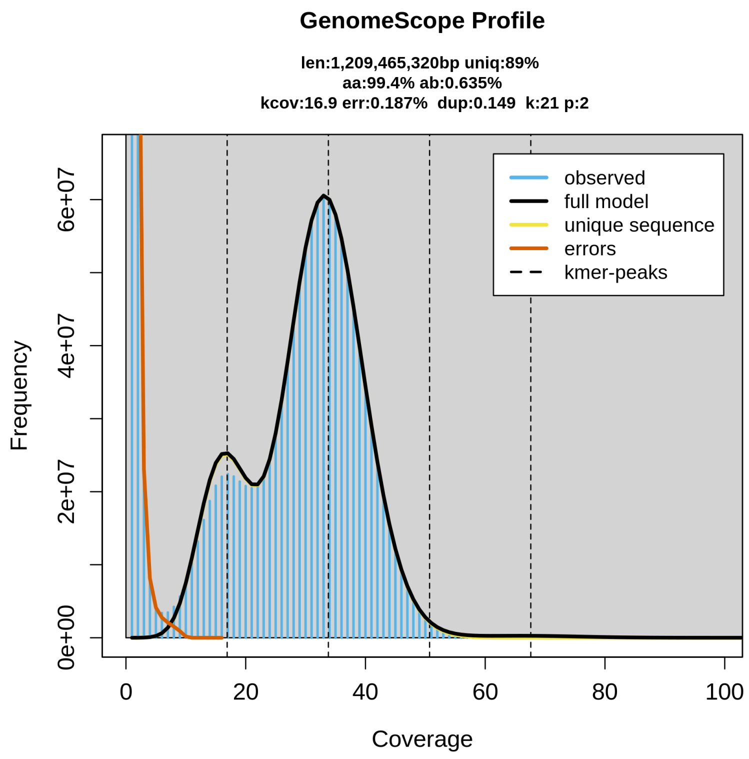

Figure S3. Genomescope2.0 21 *k*-mer profile using 21-mers for bRisTri1 generated from PacBio HiFi reads, used to estimate genome size, repetitiveness, and heterozygosity (top). The x axis represents *k*-mer coverage in the read set and the y axis represents the cumulative frequency of each *k*-mer at a given coverage.

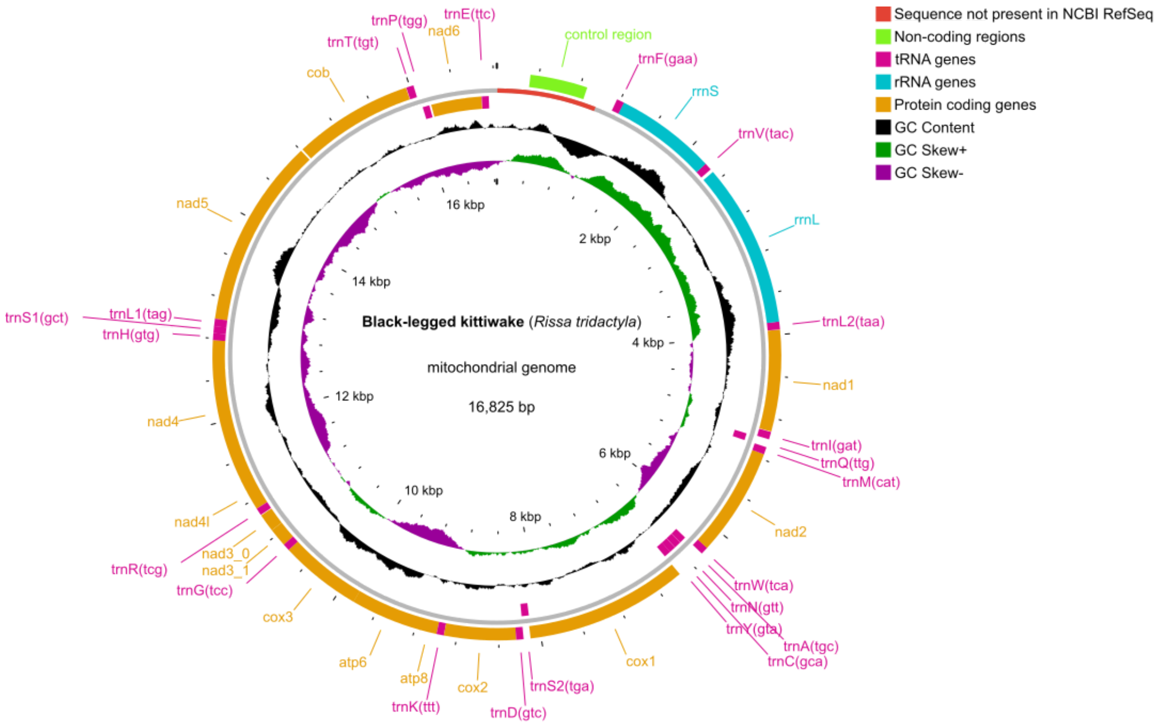

Figure S4. Graphical representation of the complete black-legged kittiwake mitogenome represented by the grey ring. The region which includes the control region that was previously not present in the NCBI reference sequence for the species is shaded in red. Genes encoded by the + strand are shown outside the grey ring, and those encoded by the - strand inside the grey ring. Intermediate rings show GC content and GC skewness.

Supplementary Tables

Table S1. Summary of RepeatMasker results showing number of elements, length occupied and % of the genome covered for each type of annotated repeats.

			number of elements	length occupied (bp)	% of sequence
Retroelements			194439	94761028	7.00
	SINEs		10177	1326896	0.10
		Penelope	158	34335	0.00
	LINEs		153680	72646310	5.37
		L2/CR1/Rex	153367	72575906	5.36
		R2/R4/NeSL	41	12241	0.00
		RTE/Bov-B	24	1511	0.00
		L1/CIN4	90	22317	0.00
	LTR elements		30582	20787822	1.54
		Gypsy/DIRS1	51	8578	0.00
		Retroviral	30307	20736965	1.53
DNA transposons			29208	4762547	0.35
		hobo-Activator	4731	835861	0.06
		Tc1-IS630-Pogo	751	133947	0.01
		Tourist/Harbinger	9272	966703	0.07
Rolling-circles			66	10706	0.00
Unclassified			3565	632416	0.05
Total interspersed repeats				100155991	7.40
Small RNA			3348	863053	0.06
Satellites			2833	445520	0.03
Simple repeats			280953	11724135	0.87
Low complexity			58937	2902794	0.21
